# Interferons in Viral Infections

**DOI:** 10.3390/v16030451

**Published:** 2024-03-14

**Authors:** Pracheta Sengupta, Saurabh Chattopadhyay

**Affiliations:** Department of Microbiology, Immunology, and Molecular Genetics, University of Kentucky College of Medicine, Lexington, KY 40536, USA; pracheta.sengupta@uky.edu

## 1. Introduction

Interferons (IFNs) are cytokines that inhibit viral replication in host cells by triggering innate immune responses through the transcriptional induction of various IFN-stimulated genes (ISGs) [1,2]. Innate immunity is the first line of defense triggered a virus entering the body. The initiation of the innate immune response is facilitated by recognizing the nucleic acids (RNA or DNA genomes and mRNA) formed during viral replication [3]. These viral nucleic acids are recognized by cellular sensor proteins known as pattern recognition receptors (PRRs), recruited during viral infection. For instance, RNA viruses are recognized by endosomal transmembrane toll-like receptors (TLR3, TLR7, and TLR8) and by cytoplasmic RIG-I-like receptors (RLRs). Meanwhile, DNA viruses are recognized by endosomal TLR9 and several cytoplasmic DNA sensors, such as cyclic GMP-AMP synthase (cGAS) [4,5,6]. TLRs are preferentially expressed in myeloid cells, such as plasmacytoid dendritic cells and macrophages, while RLRs and DNA sensors are expressed in epithelial cells and fibroblasts. Activated PRRs, in turn, trigger downstream signaling cascades that activate transcription factors such as interferon regulatory factors (IRFs) and nuclear factor kappa-light-chain-enhancer of activated B cells (NF-κB). Activating these transcription factors leads to the induction of type-I IFNs and other proinflammatory cytokines [7]. Type-I IFN binds to the dimeric type-I IFN receptor, IFNAR, which activates the JAK-STAT signaling pathway to induce interferon-stimulated genes (ISGs). Thus, the cross-talk between IFN signaling and the pathways regulating the apoptotic and inflammatory responses elicits an antiviral state in the cell [8].

As mentioned above, this interferon signaling is exhibited primarily by two types of transcription factors: IRFs and NF-κB. There are nine IRF members in mammalian cells, of which five IRFs (IRF1, IRF3, IRF5, IRF7, and IRF8) are positive regulators of type-I IFN [9]. IRF3 and IRF7 are key to IFN production in most immune cells. Abnormal production of IFNs is associated with various diseases that can damage cellular development and homeostasis. Thus, in the maintenance of homeostasis, fine-grained regulation of IFN production is controlled by a balance between activation (e.g., protein phosphorylation and ubiquitination) and deactivation (e.g., protein dephosphorylation and deubiquitination) of IRFs [9,10,11]. Another transcription factor that regulates IFN production is NF-κB, a master regulator of controlling cell proliferation, apoptosis, and viral infection. It is involved in canonical (elicited by diverse stimuli, such as PAMPs) and non-canonical (elicited by a narrow set of stimuli, such as lymphotoxin receptor ligands, CD40, RANK, and the viral latent membrane protein 1 (LMP1) of Epstein–Barr virus (EBV)) signaling cascades according to phosphorylation and polyubiquitination [12].

**Figure 1:**
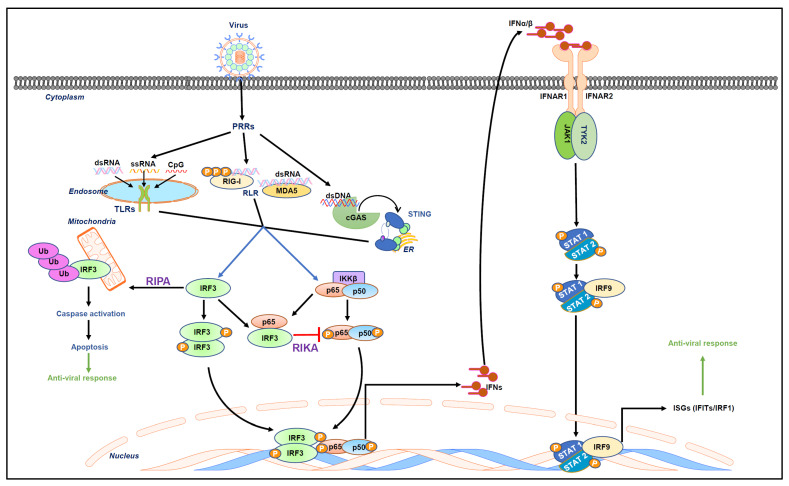
Virus-activated intracellular signaling pathways. The recognition of viral nucleic acids by cellular sensors, also known as PRRs, includes endosomal TLRs and RLRs triggering downstream signaling cascades to activate transcription factors such as IRF3 and NF-κB. These transcription factors mediate the induction of type-I IFN. Type-I IFN includes IFNα/β, which bind to IFN receptors (IFNARs) to activate the JAK/STAT signaling pathway. In this signaling pathway, the canonical signal transducers and activators of transcription (STAT)-1 and 2 dimerize with IRF9 and form a signaling complex. This binds to IFN-stimulated response elements (ISREs) to induce ISGs that elicit an antiviral state in the cell. Recently, two non-transcriptional functions of IRF3, namely RIPA and RIKA, have been uncovered. RIPA (RIG-I-like receptor-induced IRF3-mediated pathway of apoptosis) involves linear polyubiquitination of IRF3 upon viral infection, resulting in the activation of downstream caspases that apoptotically kill virus-infected cells. On the other hand, RIKA (repression of IRF3-mediated NF-κB activity) is involved in the downregulation of viral inflammation.

Type-I IFN signaling causes the rapid induction of antiviral genes known as ISGs. There are more than 300 ISGs, such as IFITs, PKR, MxA, OAS, viperin, ISG15, and TDRD7, that function as antiviral genes [13,14]. Many of these ISGs function as viral restriction factors by directly interfering with specific stages of virus replication. Some of them regulate the cellular proteins required for viral replication [1,15,16,17] (Figure 1).

## 2. Overview of the Published Articles

This Special Issue collates a variety of studies, focusing on research related to recent advancements in the field of interferon signaling during viral infection. The articles published in the issue are categorized into three different topics pertaining to the role of interferons in viral infection. The first section comprises four articles on the ongoing research performed by various groups.

Evans et al. explain the influence of the functions and regulation of interferon lambda receptor 1 (IFNLR1) isoforms on cellular responses to interferons. This publication reveals that there are three different IFNLR1 isoforms (1, 2, and 3), out of which IFNLR1 isoform 1 has the highest transcriptional expression, encoding the functions of canonical IFNL signaling. IFNLR1 isoforms 2 and 3 mainly encode the signaling of defective proteins and have lower transcriptional expressions. Their findings suggest that a low expression of IFNLR1 isoform 1 is responsible for inducing IFNL3-dependent antiviral and pro-inflammatory genes. A low expression of IFNLR1 isoform 2 could lead to the potential induction of antiviral genes but not pro-inflammatory genes. However, when treated with IFNL3, this phenotype was highly abrogated, with an increase in the expression of IFNLR1 isoform 2. Moreover, the expression of antiviral genes increases with the expression of IFNLR1 isoform 3 on treatment with IFNL3. From these findings, they concluded the differential expression of IFNLR1 isoforms could potentially influence cellular responses to both endogenous and therapeutically administered IFNLs.

Furthermore, Boeren et al. studied the activation of interferon-stimulated genes (ISGs) in Varicella zoster virus infection in human iPSC-derived neuronal cells. Their findings suggest that upon VZV infection, hiPSC neuronal cells are unable to trigger the IFN-stimulating antiviral pathway. Further studies have emphasized that upon VZV infection, neuronal cells cannot upregulate ISGs to produce an antiviral effect. Furthermore, their study indicates that hiPSC neuronal cells are unable to produce IFNα even upon its stimulation with strong synthetic inducers such as poly(dA:dT) and poly(I:C). However, when exogenous IFNα (IFNα2a) is administered at low and high doses, these cells effectively restrict the spread of VZV. At lower doses, IFNα2a has no effect on the initial infection when observed three days post-infection (dpi), while at a higher concentration, the infection is reduced significantly. At seven dpi, the effects are more prominent, with the lower dose of IFNα2a resulting in a significant reduction in eGFP+ dots arising from VZV ORF23 expression. The authors concluded that the exogenous administration of IFNα to human iPSC-derived neurons in compartmentalized chambers effectively limits the spread of VZV and significantly upregulates ISGs. As a result, the neurons adequately respond to INFα treatment, thereby underscoring its importance in early immune response, as well as highlighting the role of other glial cells in triggering innate immunity in response to VZV infection.

Next, Choi et al.’s article reports on the use of Poly6 (Hepatitis B virus-derived hexamer peptide) as an antiviral therapeutic against SARS-CoV-2 infection. Poly6 exerts an antiviral effect in an IFNI-dependent manner by inhibiting IL-6 production at both the transcriptional and translational levels, mediated by IL-10 induction.

Meanwhile, Ramnani et al. uncover the role of the ATP-binding cassette E1 (ABCE1) transporter in autophagy during viral infections. Activation of the latent, ubiquitous endoribonuclease, ribonuclease L (RNase L), in the cells produces IFN during viral infections, which regulates the degradation of the cellular and viral RNAs required during viral replication. The IFN-inducible oligoadenylate synthetase/ribonuclease L (OAS/RNase L) pathway is activated by dsRNA to synthesize unique oligoadenylates, 2-5A, that activate RNase L. The binding of 2-5A to RNAse L induces autophagy. However, the authors identified that ABCE1 inhibits RNase L activity. Cells with reduced ABCE1 levels, upon 2-5A transfection, showed an increase in RNase L activity that corresponded to the early onset of autophagy. Their findings suggest that RNAse L activity in ABCE1-depleted cells inhibits cellular proliferation and triggers the process of apoptosis. This mechanism leads to an increase in caspase 3 activity that causes the premature cleavage of Beclin 1 (autophagy protein), resulting in a switch from autophagy to apoptosis.

The next section of the issue included three review articles that outlined recent advancements in the research related to the role of IFNs in viral infection.

The first review article by Franco et al. discusses the role of IFN-induced tetratricopeptide repeat (IFIT) proteins in different pathologies. IFITs are ISGs that have been extensively studied for their antiviral activities during viral pathogenesis. However, their role in non-viral pathogenesis, such as cancer and sepsis, has also been established. In cancer, IFIT1 and IFIT3 promote metastasis, while IFIT2 exhibits the opposite effect. The role of IFITs in bacterial/fungal sepsis is still under investigation.

The next article reviews the role of the stimulator of interferon gene (STING) in the replication of herpes simplex virus (HSV). The authors (Krawczyk et al.) state that during HSV infection, dsDNA (either derived from the virus or host cells) is recognized as a PAMP that activates the STING signaling pathways. STING facilitates the activation of autophagy and the NF-κB transcription factors that are responsible for the production of IFN, which leads to antiviral responses. However, HSV, on the other hand, utilizes viral genes to suppress STING activation to persist within living organisms for longer periods and establish latency.

Glanz et al. elaborate on the transcriptional and non-transcriptional activation, post-transcriptional modification, and antiviral activity of IRF3. In addition, the article covers how SARS-CoV can antagonize IRF3 activation. IRF3 is a key transcription factor activated during viral infection. In addition to acting as a transcription factor, IRF3 activates apoptosis in virus-infected cells to restrict a virus from spreading within the host, according to the RIG-I-like receptor-induced IRF3 mediated pathway of apoptosis (RIPA). This dual role of IRF3 mediates protective mechanisms against viral infection.

Finally, Chakravarty et al.’s commentary elucidates the newly discovered anti-inflammatory function of IRF3 in RIKA (repression of IRF3-mediated NF-κB activity). In RIKA, IRF3 binds directly to the NF-κB-p65 subunit to suppress NF-κB’s induction of inflammatory genes. Mechanistically, IRF3 sequesters NF-κB in the cytosol to inhibit its nuclear translocation and subsequently target gene expression.

## 3. Conclusions

This compilation of articles sheds new light on unexplored aspects of interferon production and interferon signaling pathway activation upon viral infection. Moreover, they discuss how transcription factors, e.g., IRFs and NF-kB, that have been traditionally known to cooperatively induce overlapping genes, e.g., IFNb, can engage in other cellular functions. With these newly discovered roles of the IFN system and the multifaceted STING and IRF3 proteins, future research can only open new doors for foundational discoveries, as well as potential for therapeutics.
